# Supplementary material for: Detecting and Quantifying Changing Selection Intensities from Time-Sampled Polymorphism Data
Source: G3 (Bethesda). 2016 Feb 10;6(4):893–904. doi: 10.1534/g3.115.023200 (PMC4825659; doi:10.1534/g3.115.023200)
Supplement: Supporting Information [file supp_6_4_893__index.html]

Detecting and Quantifying Changing Selection Intensities from Time-Sampled Polymorphism Data — Supporting Information 

# Detecting and Quantifying Changing Selection Intensities from Time-Sampled Polymorphism Data

## Supporting Information for Shim *et al.*, 2016

**Files in this Data Supplement:**

- Figure S1 - Updated prior distributions of *s1*, *s2*, *CP* for a haploid population with *Ne*=100 after the constraint of trajectories segregating at change point. (.pdf, 379 KB)
- Figure S2 - Updated prior distributions of *s1*, *s2*, *CP*, *h* for a diploid population with *Ne*=50 after the constraint of trajectories segregating at change point. (.pdf, 401 KB)
- Figure S3 - ROC curves of the Bayes factor *B0,1* from the ABC model choice of a haploid population with *Ne*=1000,10000 (above) and a diploid population with *Ne*=50,5000 (below). (.pdf, 340 KB)
- Figure S4 - ABC model choice parameter estimations for 1000 pseudo-observables with a haploid population of *Ne*=1000. (.pdf, 517 KB)
- Figure S5 - ABC model choice parameter estimations for 1000 pseudo-observables with a haploid population of *Ne*=10000. (.pdf, 516 KB)
- Figure S6 - ABC model choice parameter estimations for 1000 pseudo-observables with a diploid population of *Ne*=50. (.pdf, 565 KB)
- Figure S7 - ABC model choice parameter estimations for 1000 pseudo-observables with a diploid population of *Ne*=5000. (.pdf, 592 KB)
- Figure S8 - Allele frequency of the *medionigra* morph from 1939 to 1999. (.pdf, 336 KB)
- Figure S9 - Posterior distributions for the *medionigra* morph after the ABC model choice. (.pdf, 412 KB)
