# Supplementary material for: Detecting and Quantifying Changing Selection Intensities from Time-Sampled Polymorphism Data
Source: G3 (Bethesda). 2016 Feb 10;6(4):893–904. doi: 10.1534/g3.115.023200 (PMC4825659; doi:10.1534/g3.115.023200)
Supplement: Supporting Information [file supp_g3.115.023200_FigureS2.pdf]

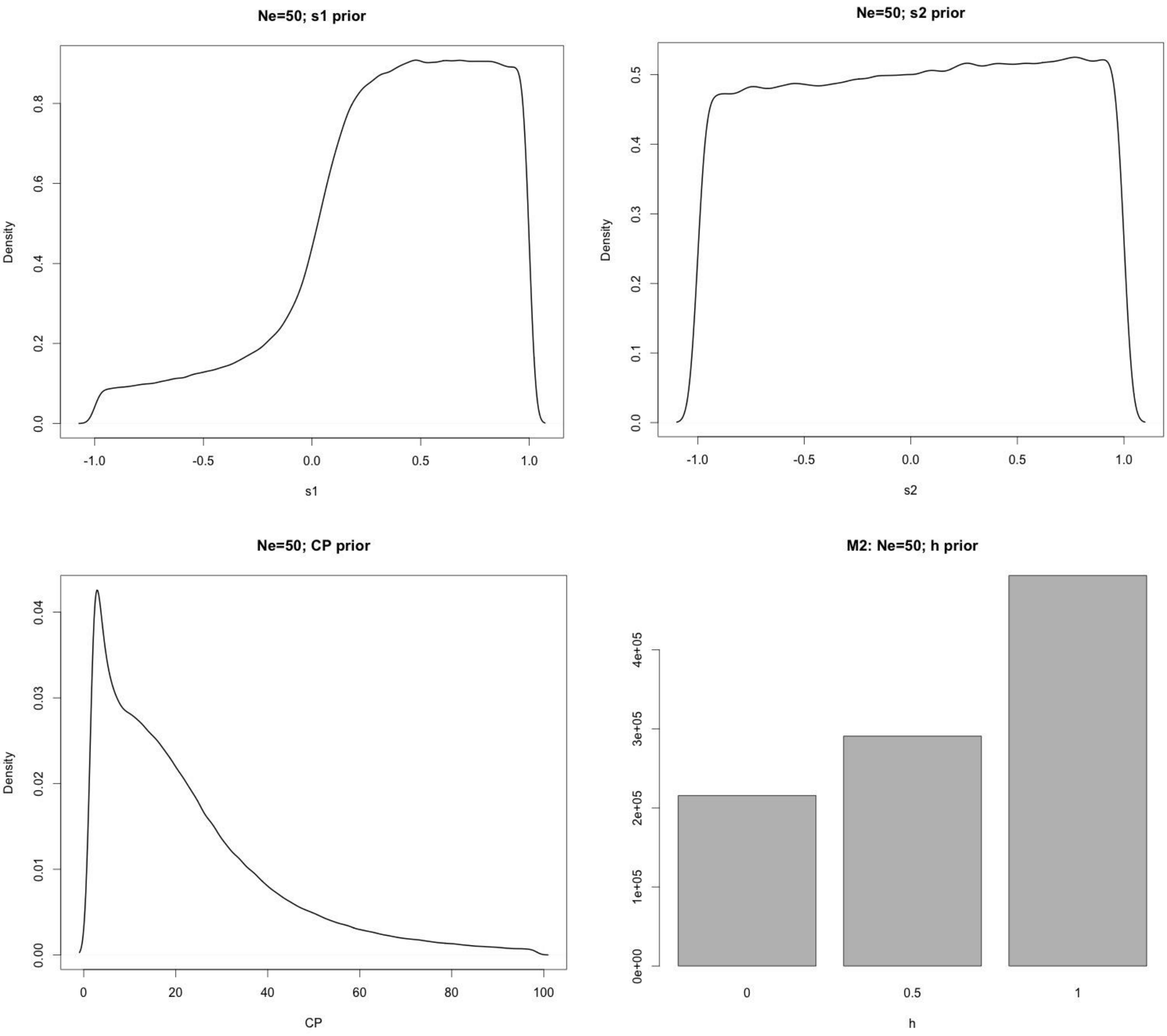

Figure S2. Updated prior distributions of *s1*, *s2*, *CP*, *h* for a diploid population with N_e=50 after the constraint of trajectories segregating at change point.
