# Supplementary material for: Detecting and Quantifying Changing Selection Intensities from Time-Sampled Polymorphism Data
Source: G3 (Bethesda). 2016 Feb 10;6(4):893–904. doi: 10.1534/g3.115.023200 (PMC4825659; doi:10.1534/g3.115.023200)
Supplement: Supporting Information [file supp_g3.115.023200_FigureS3.pdf]

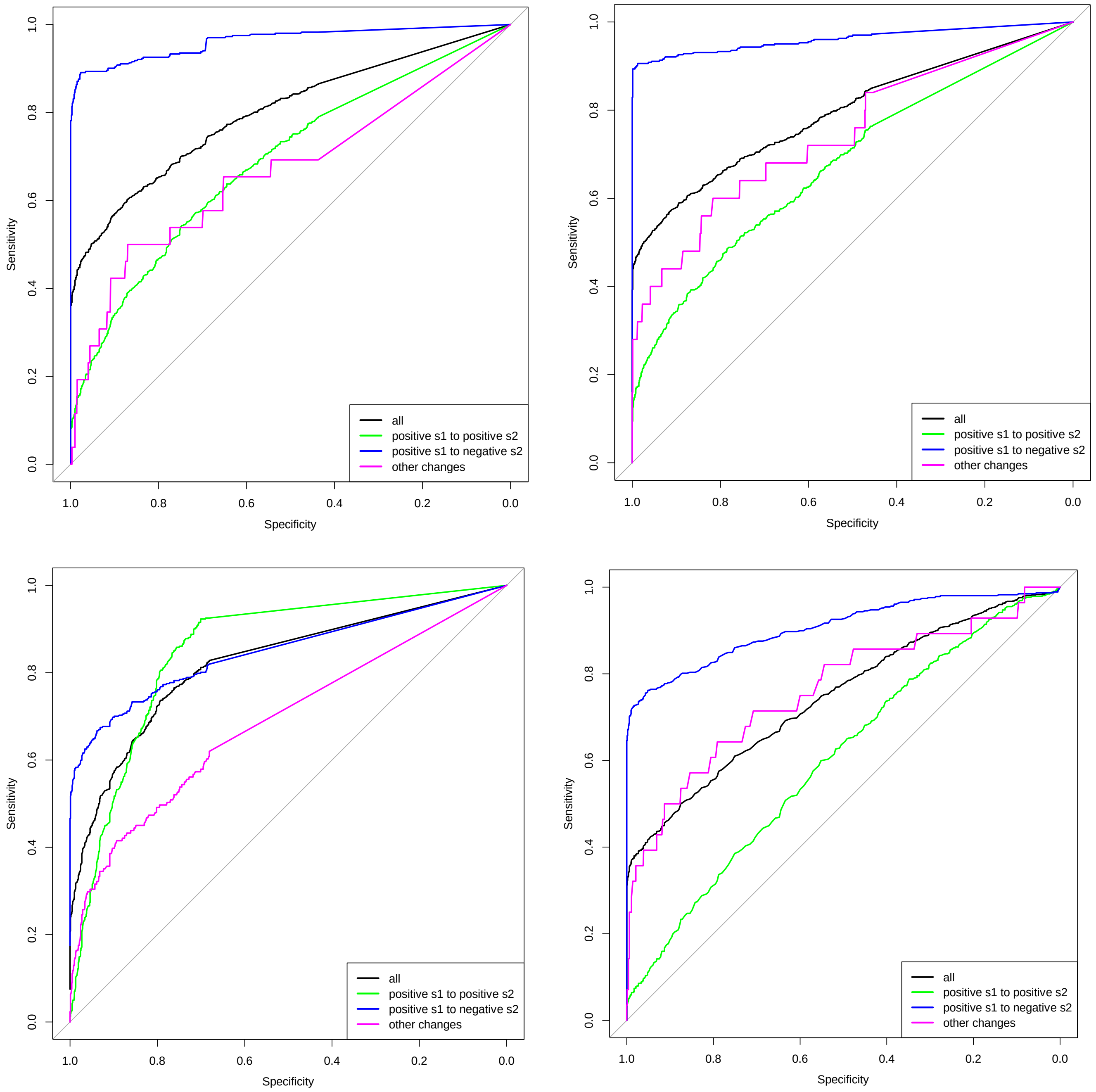

Figure S3. ROC curves of the Bayes factor $B_{0,1}$ from the ABC model choice of a haploid population with N_e=[1000,10000] (above) and a diploid population with N_e=[50,5000] (below).
