# Supplementary material for: Detecting and Quantifying Changing Selection Intensities from Time-Sampled Polymorphism Data
Source: G3 (Bethesda). 2016 Feb 10;6(4):893–904. doi: 10.1534/g3.115.023200 (PMC4825659; doi:10.1534/g3.115.023200)

Figure S7. ABC model choice parameter estimations for 1000 pseudo-observables with a diploid population of N_e=5000. Each circle is the mode of the posterior distribution from the 0.1% best simulations.

Case 1: Pseudo-observables with constant selection (A) M_0 estimates of *s* (B) M_0 estimates of *h*

A **B**

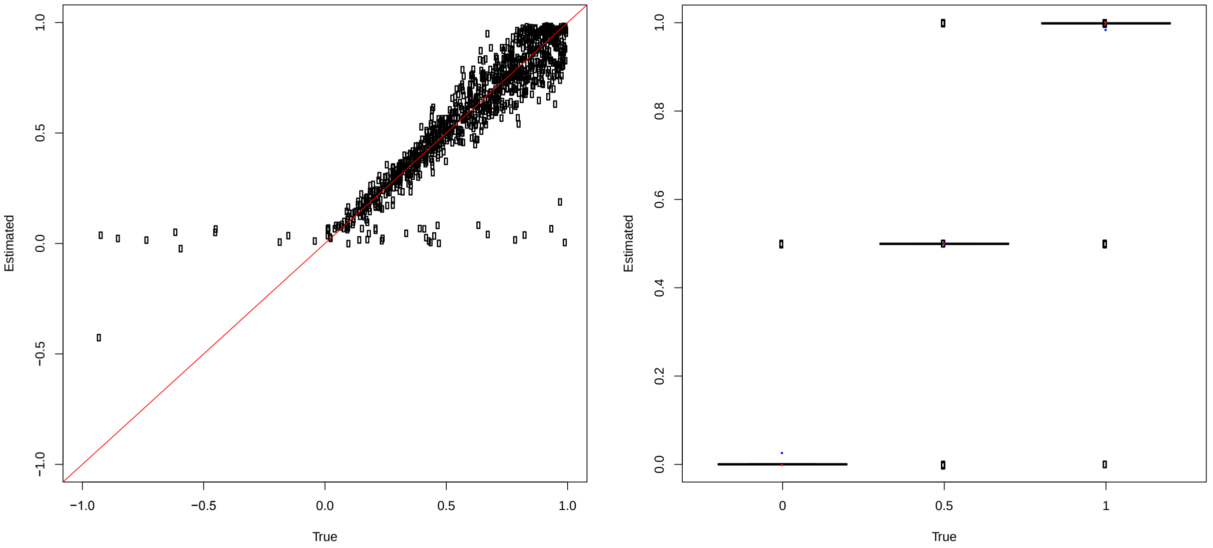

Case 2: Pseudo-observables with changing selection (C) M_1 estimates of *s1* (D) M_1 estimates of *s2* (E) M_1 estimates of *CP* (F) M_1 estimates of *h*

C **D**

E **F**

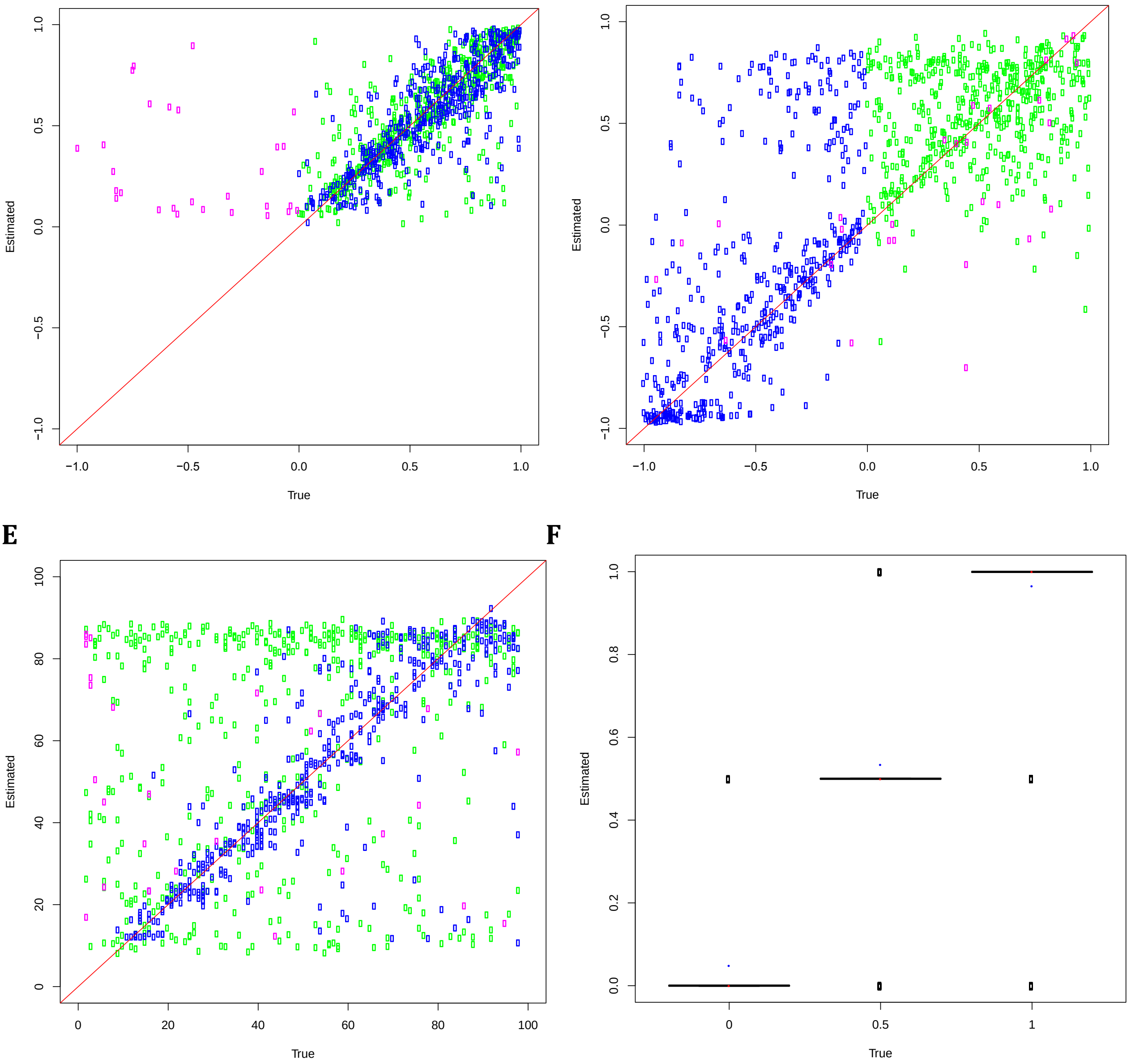

Supplement: Supporting Information [file supp_g3.115.023200_FigureS7.pdf]
