# Supplementary figures and images for: Detecting and Quantifying Changing Selection Intensities from Time-Sampled Polymorphism Data
Source: G3 (Bethesda). 2016 Feb 10;6(4):893–904. doi: 10.1534/g3.115.023200 (PMC4825659; doi:10.1534/g3.115.023200)

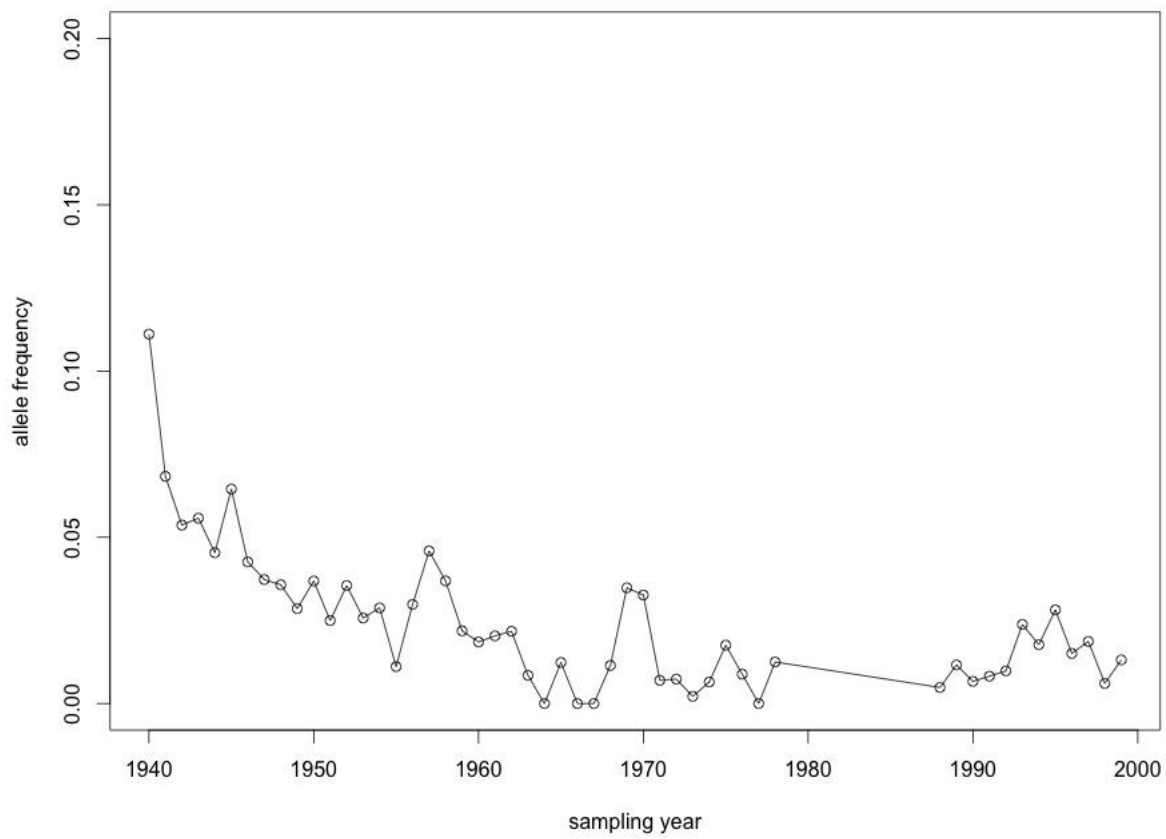

**Figure S8.** Allele frequency of the *medionigra* morph from 1939 to 1999.

Supplement: Supporting Information [file supp_g3.115.023200_FigureS8.pdf]
