# Supplementary material for: Detecting and Quantifying Changing Selection Intensities from Time-Sampled Polymorphism Data
Source: G3 (Bethesda). 2016 Feb 10;6(4):893–904. doi: 10.1534/g3.115.023200 (PMC4825659; doi:10.1534/g3.115.023200)

Figure S9. Posterior distributions for the *medionigra* morph after the ABC model choice.

N_e = 500: *s* estimated from the M_0 model

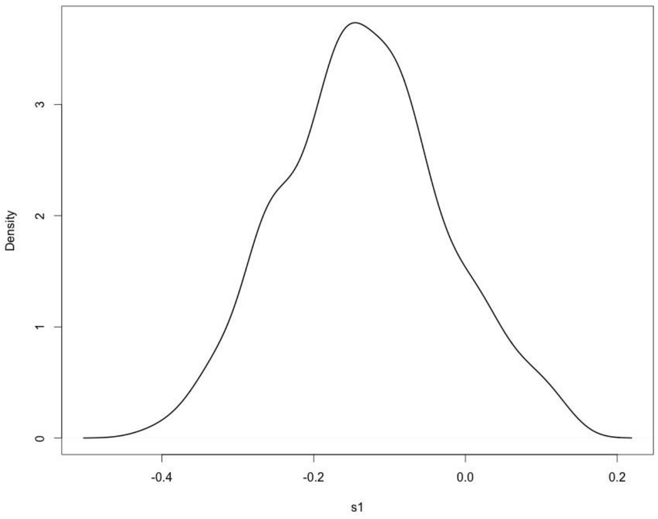

N_e = 50: *s1*, *s2*, *CP* estimated from the M_1 model

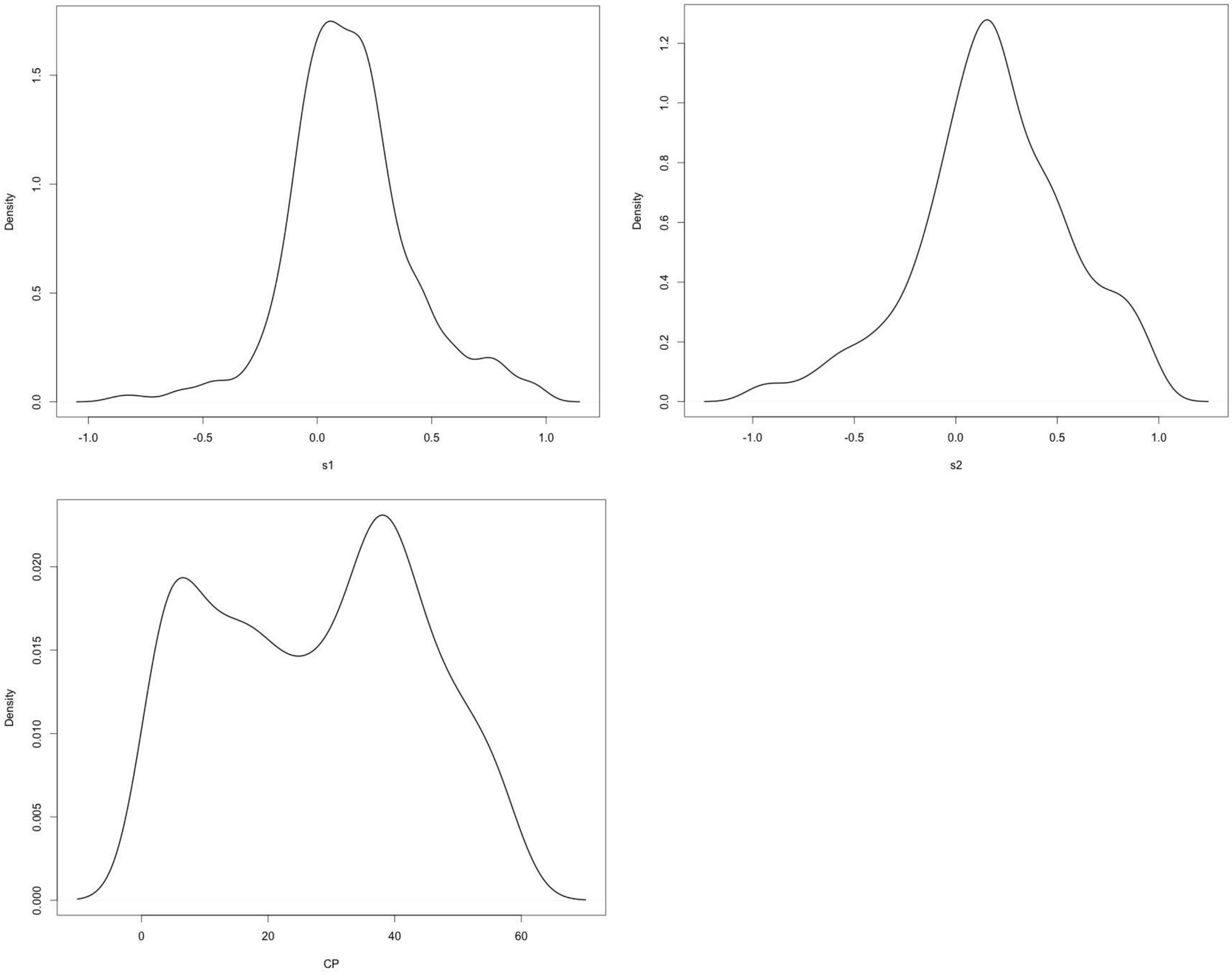

Supplement: Supporting Information [file supp_g3.115.023200_FigureS9.pdf]
